# Supplementary material for: An open-source framework for large-scale, flexible evaluation of biomedical text mining systems
Source: J Biomed Discov Collab. 2008 Jan 29;3:1. doi: 10.1186/1747-5333-3-1 (PMC2276192; doi:10.1186/1747-5333-3-1)
Supplement: Additional file 1 — Evaluation platform source code. The additional file contains the software evaluation framework discussed in this paper. The most current release can be downloaded from SourceForge.net [1]. See the accompanying README file for instructions on installation and use. Also included in the distribution is a collection of UIMA wrappers for some commonly used BioNLP tools and annotated corpora. [file 1747-5333-3-1-S1.zip › bionlp_uima_repository-1.0.1/docs/api/src-html/edu/uchsc/ccp/util/nlp/KeyValuePair.html]

```
001    /*
002     *
003     * The Original Code is UCHSC Utilities.
004     *
005     * The Initial Developer of the Original Code is University of Colorado.  
006     * Copyright (C) 2005.  All Rights Reserved.
007     *
008     * UCHSC Utilities was developed by the Center for Computational Pharmacology
009     * (http://compbio.uchsc.edu) at the University of Colorado Health 
010     *  Sciences Center School of Medicine with support from the National 
011     *  Library of Medicine.  
012     *
013     *
014     * Contributor(s):
015     *   William A. Baumgartner <william.baumgartner@uchsc.edu> (Original Author)
016     *   
017     * Created: September 2005
018     * Dependencies:
019     * ToDo: 
020     *      This class should be generalized so that the key and value can be Objects, allowing the compareTo method to be overrided
021     * Changes:
022     */
023    package edu.uchsc.ccp.util.nlp;
024    
025    /**
026     * This class is a utility class used for interacting with HashMaps, and potentially and application requiring Key/Value pairings.
027     * @author William A. Baumgartner, Jr.
028     *
029     */
030    public class KeyValuePair implements Comparable {
031        private String key;
032            private int value;
033            
034        /**
035         * Construct a new KeyValuePair with the given input arguments.
036         * @param key the key
037         * @param value the value
038         */
039            public KeyValuePair(String key, int value) {
040                    this.key = key;
041                    this.value = value;
042            }
043            
044        /**
045         * Returns the key
046         * @return a string containing the key
047         */
048            public String getKey() {
049                    return key;
050            }
051        
052        /**
053         * Sets the key
054         * @param key a string containing the key
055         */
056            public void setKey(String key) {
057                    this.key = key;
058            }
059        
060        /**
061         * Returns the value
062         * @return an int value
063         */
064            public int getValue() {
065                    return value;
066            }
067        
068        /**
069         * Sets the value
070         * @param value an int value
071         */
072            public void setValue(int value) {
073                    this.value = value;
074            }
075    
076            /* (non-Javadoc)
077             * @see java.lang.Comparable#compareTo(java.lang.Object)
078             * see Comparator example: http://www.onjava.com/lpt/a/3286
079             */
080        /**
081         * This method enables comparison between two KeyValuePairs. 
082         * @param anotherKVP the KeyValuePair to compare with
083         */
084            public int compareTo(Object anotherKVP) {
085                    if (!(anotherKVP instanceof KeyValuePair))
086                          throw new ClassCastException("A KeyValuePair object expected.");
087                int anotherKVPValue = ((KeyValuePair) anotherKVP).getValue();
088                String anotherKPVKey = ((KeyValuePair) anotherKVP).getKey();
089                
090                // sort by value (the number of times the token was seen) then alphabetically by key (the token)
091                if (anotherKVPValue != this.value) {
092                return  anotherKVPValue - this.value;
093                } else {
094                    return (this.key).compareTo(anotherKPVKey);
095                }
096            }
097    }
```
